# Neural precursor cells are decreased in the hippocampus of the delayed carbon monoxide encephalopathy rat model

**DOI:** 10.1038/s41598-021-85860-9

**Published:** 2021-03-18

**Authors:** Shinichiro Ochi, Keisuke Sekiya, Naoki Abe, Yu Funahashi, Hiroshi Kumon, Yuta Yoshino, Tasuku Nishihara, Shuken Boku, Jun-ichi Iga, Shu-ichi Ueno

**Affiliations:** 1 Department of Neuropsychiatry, Molecules and Function, Ehime University Graduate School of Medicine, Shitsukawa, Toon, Ehime 791-0295 Japan; 2 Department of Anesthesia and Perioperative Medicine, Ehime University Graduate School of Medicine, Shitsukawa, Toon, Ehime 791-0295 Japan; 3 Department of Neuropsychiatry, Faculty of Life Sciences, Kumamoto University, 1-1-1 Honjo, Chuo-ku, Kumamoto, 860-8556 Japan

**Keywords:** Neuroscience, Diseases of the nervous system, Neurogenesis

## Abstract

The pathophysiology of delayed carbon monoxide (CO) encephalopathy remains unclear. In this study, the effects of CO exposure on the dentate gyrus (DG) were investigated in a Wistar rat model by histochemical and molecular methods. Model rats showed significant cognitive impairment in the passive-avoidance test beginning 7 days after CO exposure. Immunohistochemistry showed that compared to the control, the cell number of SRY (sex-determining region Y)-box 2 (SOX2)^+^/brain lipid binding protein (BLBP)^+^/glial fibrillary acidic protein (GFAP)^+^ cells in the DG was significantly less, but the number of SOX2^+^/GFAP^−^ cells was not, reflecting a decreased number of type 1 and type 2a neural precursor cells. Compared to the control, the numbers of CD11b^+^ cells and neuron glial antigen 2^+^ cells were significantly less, but the number of SOX2^−^/GFAP^+^ cells was not. Flow cytometry showed that the percent of live microglial cells isolated from the hippocampus in this CO rat model was significantly lower than in controls. Furthermore, mRNA expression of fibroblast growth factor 2 and glial cell-derived neurotrophic factor, which are neurogenic factors, was significantly decreased in that area. We conclude that, in this rat model, there is an association between delayed cognitive impairment with dysregulated adult hippocampal neurogenesis and glial changes in delayed CO encephalopathy.

## Introduction

Carbon monoxide (CO) is a non-irritant, colorless, tasteless, and scentless toxic gas. CO exposure to a concentration of higher than 100 ppm is toxic to humans in room air, because the affinity of hemoglobin for CO is about 200 times higher than that of oxygen^1^. Therefore, CO binds rapidly to hemoglobin and leads to the genesis of carboxyhemoglobin, which causes CO poisoning, resulting in severe hypoxia and cell injury. Furthermore, CO also binds to other heme proteins, such as mitochondrial cytochrome C oxidase (COX). As a result of binding and inhibiting COX, CO inhibits mitochondrial respiration and causes hypoxic conditions. Furthermore, inhibition of mitochondrial function increases reactive oxygen species (ROS) and heme oxygenase-1 (HO-1). HO-1 produces CO from free heme. On the other hand, CO activates neutrophils via activation of platelets. Activation of neutrophils causes release of myeloperoxidase and proteases that damage cells and lipid peroxidation via generating ROS. Furthermore, myelin basic protein is peroxidated and causes neurological damage via proliferation of lymphocytes and activation of microglia. Hypoxia and inhibition of mitochondria from CO also cause neurological damage via glutamate release^2^. Therefore, CO poisoning causes various manifestations through these complex mechanisms. CO poisoning has both acute and delayed stages. The symptoms of acute CO poisoning depend on the concentration and duration of CO exposure and are non-specific, including headaches, dyspnea, and nausea. In severe cases, loss of consciousness, coma, and even death can develop^3^. On the other hand, delayed CO poisoning, also called “delayed CO encephalopathy”, can develop 2 to 4 weeks after recovery from acute CO poisoning^4^. Around 10 to 30% of patients with delayed CO encephalopathy have neuropsychiatric symptoms such as depression, anxiety, parkinsonism, cognitive impairment, and psychosis^5^. These neuropsychiatric symptoms are sometimes continuous and severely affect the quality of life for a long time. Generally, administration of 100% oxygen by hyperbaric oxygen (HBO) is used as a treatment for acute CO poisoning. A meta-analysis reported that HBO may reduce the occurrence of delayed neuropsychiatric symptoms^6^. However, the efficacy and mechanism of HBO for treatment of delayed CO encephalopathy-associated neuropsychiatric symptoms remain unclear, and little evidence for other treatments is available. Therefore, clarification of the pathophysiology of delayed CO encephalopathy and establishment of novel treatments based on the pathophysiology are needed.

Some previous studies have investigated the pathophysiology of delayed CO encephalopathy. For example, mice exposed to CO (15 s at a rate of 35 ml/min of pure CO gas) showed delayed cognitive impairment, delayed neural cell death in hippocampal CA1 cells, and dysfunctions of acetylcholinergic neurons in the frontal cortex and striatum after 7 days^7^. Rats exposed to CO (1000 ppm CO for 40 min and then 3000 ppm for 20 min) showed no cognitive impairment, but they showed transient degradation of myelin basic protein in the hippocampus after 14 days^8^. Rats exposed to CO by peritoneal injection (100 ml/kg of pure CO gas the first time and 50 ml/kg of pure CO gas 3 times with a 4-h interval) showed time-dependent, significantly increased levels of malondialdehyde, a marker of lipid peroxidation, in the serum and cerebral cortices. Furthermore, the levels of glutathione, the activities of glutathione peroxidase and glutathione reductase, and the amount of anti-reactive oxygen species were significantly decreased, indicating a reduced antioxidative status, for up to 21 days^9^. Rats exposed to CO (1000 ppm CO for 40 min and then 3000 ppm for 20 min) also showed time-dependent degradation of myelin basic protein and altered numbers of lymphocytes and microglia in the brain^10^. However, these results are insufficient to explain the pathophysiology of delayed CO encephalopathy.

To elucidate the effects of delayed CO encephalopathy, we previously established a rat model of delayed CO encephalopathy and showed that CO exposure causes delayed cognitive impairment and hippocampal cell death in the dentate gyrus (DG)^11^, where adult neurogenesis occurs. Adult neurogenesis in the DG plays important roles in not only the developmental process of cognitive function, but also the pathophysiology of psychiatric disorders such as major depressive disorder and the mechanisms of action of psychotropic drugs^12–15^. We also showed that mRNA expression of several neurotrophic factors is decreased in the hippocampus 7 days after CO exposure^16^. Furthermore, previous studies including ours showed that the number of microglial cells and the level of glial fibrillary acidic protein (GFAP), a marker of astrocytes, are decreased 7 days after CO exposure^16, 17^. In addition, both astrocytes and microglial cells play a role in hippocampal neurogenesis, even in adults^18, 19^. These findings suggest that adult neurogenesis in the DG and glial cells, such as astrocytes and microglial cells, may be associated with the pathophysiology of delayed CO encephalopathy. However, to the best of our knowledge, no previous study has focused on the delayed effects of CO exposure on neural precursor cells in the adult DG. Thus, this study investigated the effects of CO exposure on neural precursor cells and glial cells in the granular cell layer and subgranular zone in the adult DG 21 days after CO exposure to elucidate the pathophysiology of delayed CO encephalopathy.

## Results

### Effects of CO exposure on the cognitive function of rats

**Figure 1:**
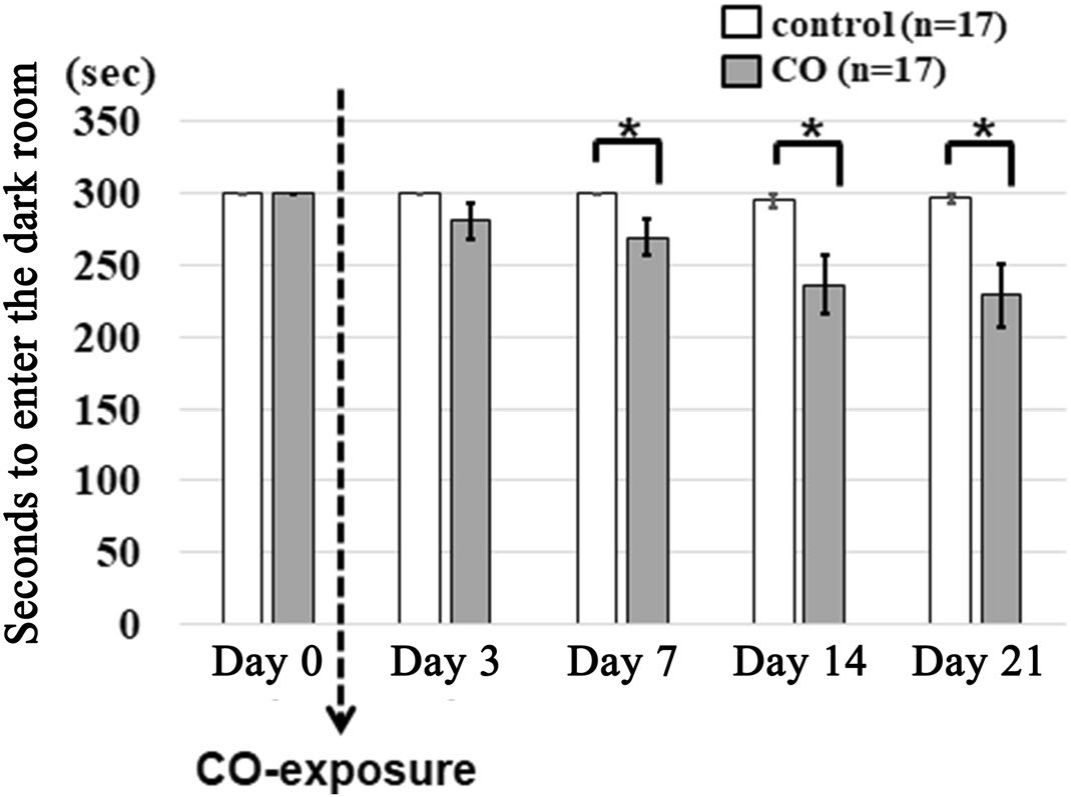
Changes in passive avoidance test latencies. The latencies in the passive avoidance test are significantly shorter in the CO rats than in controls from day 7. *p < 0.05.

It was observed that, compared to controls, the activity of all rats exposed to CO was not different 24 h after CO exposure. First, the effects of CO exposure on the cognitive function of rats were examined with the passive-avoidance test. The step-through latencies in the passive-avoidance test in the CO rats were significantly shorter than in the controls on day 7 (300.0 ± 0.0 vs. 269.2 ± 12.8, *p* = 0.029), day 14 (294.8 ± 5.0 vs. 236.0 ± 20.5, *p* = 0.036), and day 21 (296.6 ± 3.4 vs. 229.0 ± 22.4, respectively, *p* = 0.013) after CO poisoning (Fig. 1). These results suggest that the procedure of CO exposure induced delayed cognitive impairment in the rats and may be applicable as a model of delayed CO encephalopathy.

### Effects of CO exposure on the numbers of neural precursor cells and glial cells

**Figure 2:**
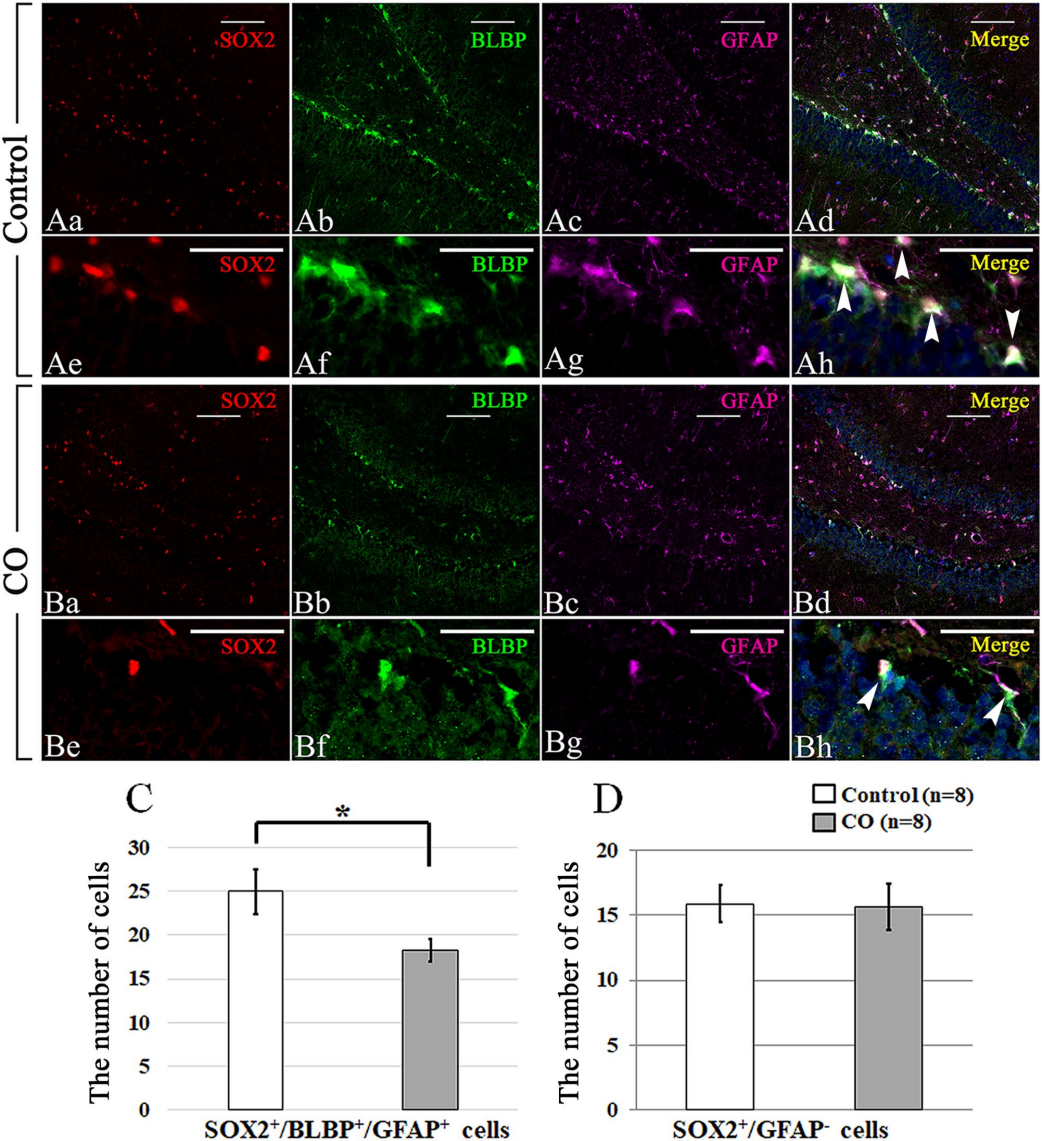
Immunohistochemical analyses of the dentate gyrus 21 days after CO exposure, and the number of SOX2^+^/GFAP^+^/BLBP^+^ and SOX2^+^/GFAP^−^ cells in the granular layer of the dentate gyrus. (**A**) Immunohistochemical analyses were performed using antibodies against SOX2 (red), BLBP (green), and GFAP (purple) for controls (**Aa**–**h**) at 21 days. Arrow head (**B**) Immunohistochemical analyses were performed using antibodies against SOX2 (red), BLBP (green), and GFAP (purple) for the CO rats (**Ba**–**h**) at 21 days after CO exposure. (**C**) The number of SOX2^+^/GFAP^+^/BLBP^+^ cells is significantly lower in the CO rats 21 days after CO exposure than in the controls. (**D**) The number of SOX2^+^/GFAP^−^ cells is not significantly different between CO rats and controls. Nuclear staining is performed using DAPI. Arrowheads show SOX2^+^/GFAP^+^/BLBP^+^ cells. Scale bars = 100 µm (**Aa**–**h**, **Ba**–**h**). Values are expressed as mean ± SEM. *p < 0.05.

**Figure 3:**
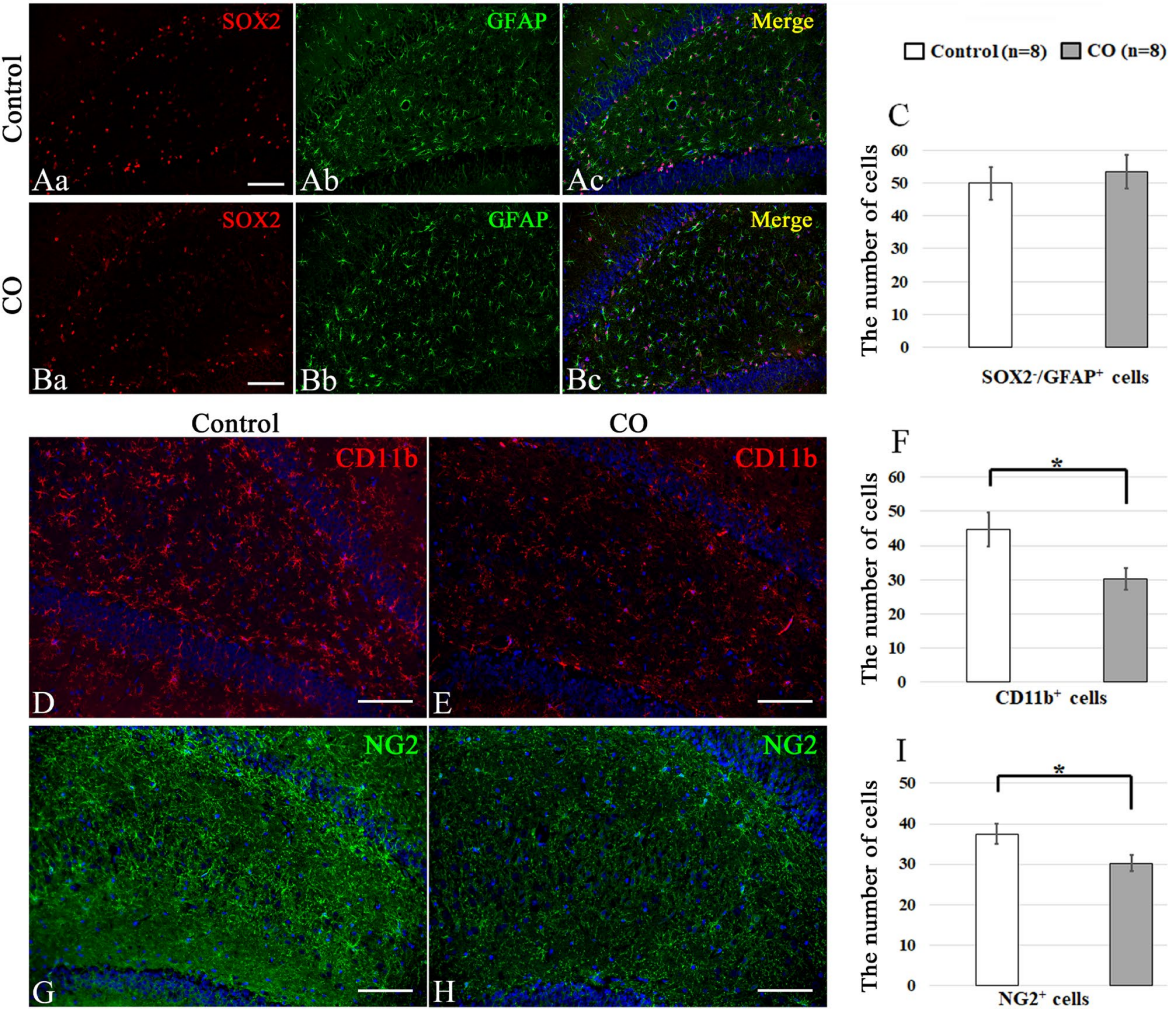
Immunohistochemical analyses of the dentate gyrus 21 days after CO exposure, and the number of SOX2^−^/GFAP^+^, CD11b^+^, and NG2^+^ cells in the dentate gyrus. (**A**) Immunohistochemical analyses were performed using antibodies against SOX2 (red) and GFAP (green) for controls (**Aa**–**c**) at 21 days. (**B**) Immunohistochemical analyses were performed using antibodies against SOX2 (red) and GFAP (green) for the CO rats (**Ba**–**c**) at 21 days after CO exposure. (**C**) The number of SOX2^−^/GFAP^+^ cells is not significantly different between CO rats and controls. (**D**) Immunohistochemical analyses were performed using antibodies against CD11b (red) for controls at 21 days. (**E**) Immunohistochemical analyses were performed using antibodies against CD11b (red) for the CO rats at 21 days after CO exposure. (**F**) The number of CD11b^+^ cells is significantly lower in the CO rats 21 days after CO exposure than in the controls. (**G**) Immunohistochemical analyses were performed using antibodies against NG2 (green) for controls at 21 days. (**H**) Immunohistochemical analyses were performed using antibodies against NG2 (green) for the CO rats at 21 days after CO exposure. Nuclear staining was performed using DAPI solution. (**I**) The number of NG2^+^ cells is significantly lower in the CO rats 21 days after CO exposure than in the controls. Scale bars = 100 µm (**Aa**–**c**, **Ba**–**c**, **D**, **E**, **G**, **H**). Values are expressed as mean ± SEM. *p < 0.05.

Next, the effects of CO exposure on the numbers of neural precursor cells and glial cells in the DG were examined by immunohistochemistry (IHC). The average total number of cells (n = 8/group) is shown in Fig. 2. SOX2^+^/BLBP^+^/GFAP^+^ cells (type 1 and type 2a neural precursor cells, Fig. 2A,B) and SOX2^+^/GFAP^−^ cells (type 2b neural precursor cells, Fig. 2A,B) were observed in the DG of both controls and CO rats. The number of SOX2^+^/BLBP^+^/GFAP^+^ cells was significantly less in CO rats than in controls (Fig. 2C), but the number of SOX2^+^/GFAP^−^ cells was similar in both groups (Fig. 2D). SOX2^−^/GFAP^+^ cells (astrocytes, Fig. 3A,B) were observed in the DG of both controls and CO rats, and the numbers of SOX2^−^/GFAP^+^ cells were not significantly different between controls and CO rats (Fig. 3C). CD11b^+^ cells (microglial cells, Fig. 3D,E) and the numbers of CD11b + cells were significantly less in CO rats than in controls (Fig. 3F). NG2^+^ cells (oligodendrocyte precursor cells, Fig. 3G,H) were observed in the DG of both controls and CO rats. and the numbers of NG2^+^ cells were significantly less in CO rats than in controls (Fig. 3I). These results suggest that CO exposure may decrease the numbers of type 1 and type 2a neural precursor cells, microglial cells, and oligodendrocyte precursor cells, but not astrocytes.

### Detailed analysis of the effects of CO exposure on the number of microglial cells

**Figure 4:**
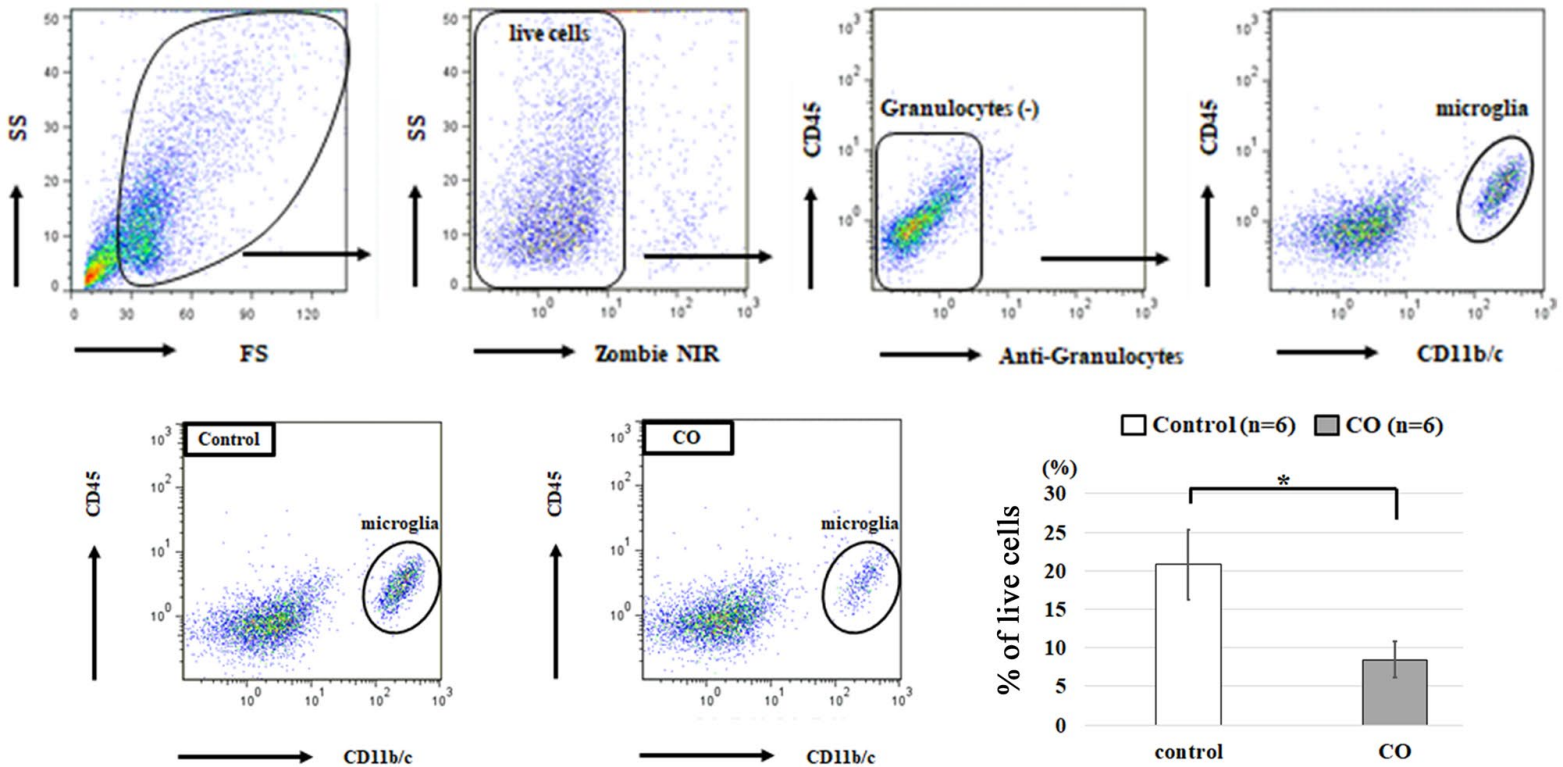
Analyses of microglial cells in the hippocampus at day 21 with flow cytometry (FCM). (**A**) The gating strategy of FCM for analyses of microglial cells. Live cells were gated based on forward scatter (FS) and side scatter (SS) values to remove debris, followed by exclusion of dead cells using Zombie NIR. Live cells were analyzed by separating granulocytes using CD45-PE and granulocytes-FITC. Granulocytes were identified as CD45^hi^/granulocyte^+^ cells. Cell populations excluding granulocytes were analyzed by separating microglial cells using CD45-PE and CD11b/c-PE/cy7. Microglial cells were identified as CD45^+^/CD11bc^+^ cells. (**B**) Microglial cells identified as CD45^+^/CD11bc^+^ cells are decreased in the CO rats compared with controls. (**C**) The percent of microglial cells among live cells is significantly decreased in the CO group. Values are expressed as mean ± SEM. *p < 0.05.

The procedure of isolation of microglial cells from brain tissues and cell counting of isolated microglia with FCM is well-established, but that of astrocytes, oligodendrocyte precursor cells, and neural precursor cells is not. Therefore, FCM was used, and detailed analysis of the effects of CO exposure on the number of microglia was performed. The gating strategy is shown in Fig. 4A. Detailed analysis with FCM showed that the percent of live microglia was significantly less in CO rats than in controls (6.5% ± 4.5% vs. 20.8% ± 12.1%, respectively, *p* = 0.022, Fig. 4B,C), similar to the IHC analysis.

### Effects of CO exposure on mRNA expression of neurogenic factors

**Figure 5:**
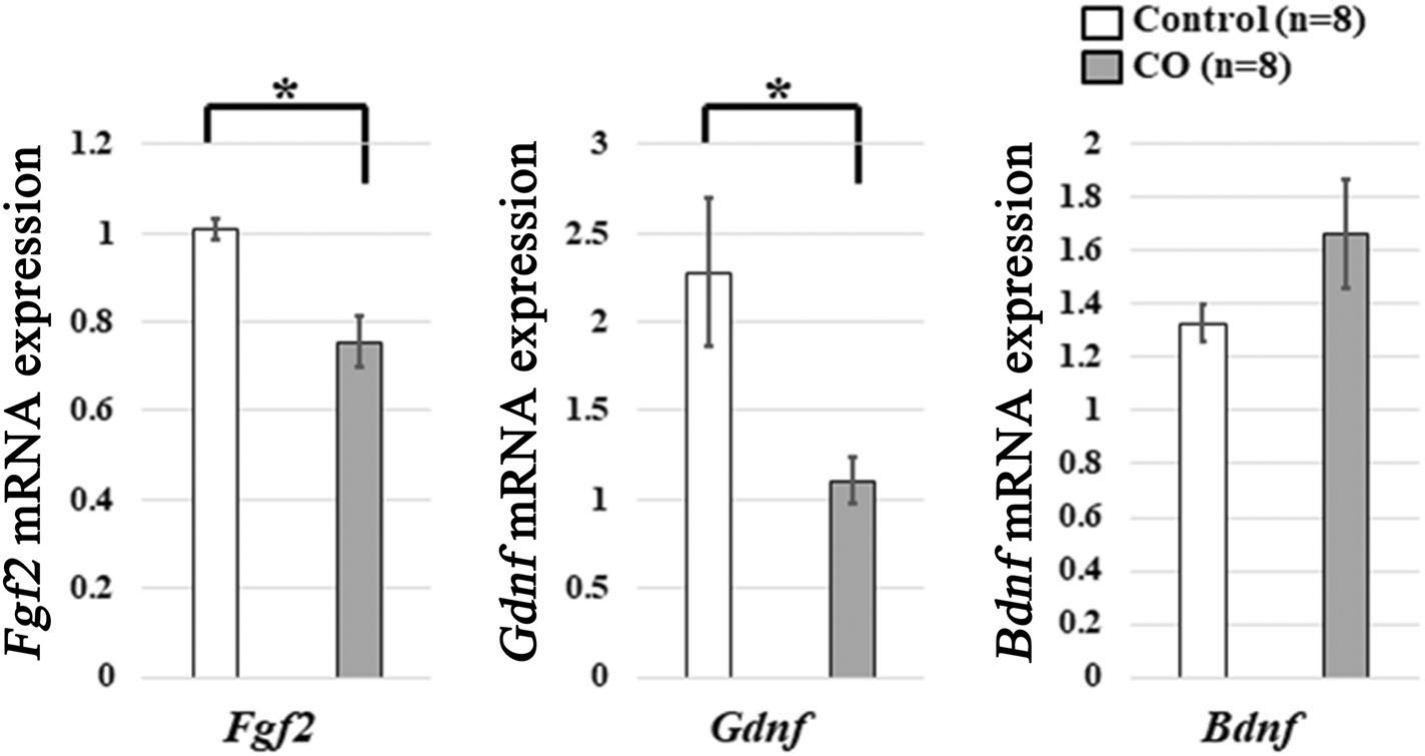
mRNA expression of neurotropic factors in neural precursor cells and glial cells. The mRNA expression of *Fgf2* and *Gdnf* is significantly lower in the CO rats 21 days after CO exposure than in the controls, and the mRNA expression of *Bdnf* is not significantly different between CO rats and controls. Values are expressed as mean ± SEM. *p < 0.05.

To investigate the mechanism underlying the negative effect of CO exposure on neural precursor cells, the effects of CO exposure on mRNA expression of common neurogenic factor genes in adult hippocampal neurogenesis, such as *Bdnf*, *Fgf2*, and *Gdnf*, were examined with quantitative RT-PCR. The mRNA expression of *Fgf2* (*p* = 0.002) and *Gdnf* (*p* = 0.002) was significantly less in CO rats than in controls, but no difference was seen for *Bdnf* (*p* = 0.13) (Fig. 5). These results suggest that CO exposure may decrease the expression of FGF2 and GDNF in the adult DG.

## Discussion

This study demonstrated the delayed effects of CO exposure on neural precursor cells and glial cells in the adult DG using our rat model to elucidate the pathophysiology of delayed CO encephalopathy. The result of the passive avoidance test suggested that the effect on cognitive function of CO poisoning could be greater in the chronic phase, at least 21 days, than in the acute phase. As is well known, adult hippocampal neurogenesis plays a role in cognitive functions^20, 21^, and glial cells support adult hippocampal neurogenesis^22^. Therefore, the present results suggest that the CO-induced decrease in adult hippocampal neurogenesis may be involved in the cognitive impairment associated with delayed CO encephalopathy.

CO exposure decreased the number of SOX2^+^/BLBP^+^/GFAP^+^ cells, but not SOX2^+^/GFAP^−^ cells or SOX2^−^/GFAP^+^ cells, in the adult DG. In the differentiation stages of adult hippocampal neurogenesis, SOX2^+^/BLBP^+^/GFAP^+^ cells correspond to type 1 neural precursor cells, which are the radial glia-like stem cells, and type 2a neural precursor cells, which can differentiate into more glial-like cells and are multipotent, and SOX2^+^/GFAP^−^ cells correspond to Type 2b neural precursor cells, which can differentiate only into neurons^19^. In addition, CO exposure decreased the number of oligodendrocyte precursor cells, which are also multipotent, as are type 1/type 2a neural precursor cells^23, 24^. Therefore, the present results suggest that CO exposure may decrease multipotent precursor cells in the adult DG.

A previous study showed that GFAP expression decreases 7 days after CO exposure^17^. The present study showed that *Gfap* mRNA expression decreased even 21 days after CO exposure (Supplemental Figure 1). Therefore, it was first presumed that CO exposure may decrease the number of astrocytes in the DG. However, as described above, CO exposure did not alter the number of astrocytes in the DG, which was confirmed by the results that the expressions of other markers for astrocytes such as *S100b*, *Glast*, and *Glt*-1 were not decreased by CO exposure (Supplemental Figure 1). In the present study, the intensity of GFAP was not investigated by immunohistochemistry, and the difference in SOX2^−^/GFAP^+^ cells between controls and CO rats might represent the change in activation of astrocytes. However, this discrepancy between GFAP expression and the number of astrocytes may be derived from GFAP expression in neural precursor cells. Type 1 neural precursor cells express GFAP well, and type 2a neural precursor cells express GFAP to varying degrees. In addition, the present results showed that CO exposure decreased the number of type 1 and type 2a neural precursor cells. Therefore, the CO exposure-induced decrease in GFAP expression may reflect a decrease in type 1 and type 2a neural precursor cells.

CO exposure did not alter the number of astrocytes. On the other hand, CO exposure decreased mRNA expression of *Fgf2* and *Gdnf*, but not *Bdnf*. Astrocytes play a role in adult hippocampal neurogenesis^22^, and astrocytes are the sources of neurogenic factors such as BDNF, FGF2, and GDNF^25^. Therefore, these findings suggest that CO exposure may have no effect on the number of astrocytes, but that exposure decreases the expression of astrocyte-derived FGF2 and GDNF, which may lead to CO exposure-induced decreases in multipotent precursor cells.

A previous study showed that antidepressants, such as tricyclic antidepressants (TCAs), selective serotonin reuptake inhibitors (SSRIs), and serotonin noradrenaline reuptake inhibitor (SNRIs), increased the expression of FGF2 in primary cultured astrocytes^25^. In addition, this TCA-induced increase in FGF2 is required for the effects of TCA on increasing the proliferation of neural precursor cells derived from the adult rat DG^26^. Taken together, these results suggest the possibility that TCAs can restore the CO exposure-induced decrease in neural precursor cells via increasing the expression of FGF2 by astrocytes. This hypothesis is expected to lead to the development of treatment for the cognitive impairment that is associated with delayed CO encephalopathy. We will perform further investigation to test this hypothesis as a next step.

Although TCAs also increase the expression of GDNF in primary cultured astrocytes^25^, no TCA-induced increase in GDNF was involved in the effects of these drugs on increasing the proliferation of neural precursor cells derived from the adult rat DG^26^. In addition, GDNF has no significant effects on the proliferation or survival of neural precursor cells derived from the adult rat DG^26^. On the other hand, GDNF increases the differentiation of neural precursor cells into astrocytes^27^. Therefore, the decrease in GDNF induced by CO exposure may not be involved in the decrease in neural precursor cells induced by CO exposure. On the other hand, GDNF has anti-apoptotic effects on neurons^28, 29^. Therefore, the decrease in GDNF induced by CO exposure may be involved in cognitive impairment by affecting neurons.

The results of both IHC and FCM analyses showed that CO exposure decreased the number of microglial cells. Activated microglial cells are considered to have negative effects on adult hippocampal neurogenesis because these cells secrete inflammatory factors, such as tumor necrosis factor-α, interleukin-1β, and interleukin-6, which induce apoptosis in neural precursor cells^30, 31^. However, recent studies have shown that these inflammatory factors can increase the proliferation of neural precursor cells depending on the conditions^32, 33^. Therefore, the decrease in microglial cells induced by CO exposure may be involved in the decrease in neural precursor cells induced by CO exposure. However, the relationship between microglial cells and adult hippocampal neurogenesis remains unclear, and further investigation is required to clarify the functional significance of the decrease in microglial cells in the pathophysiology of delayed CO encephalopathy. To clarify the effect of CO in greater detail, staining with Ki67 and/or bromodeoxyuridine (BrdU) is needed to analyze cell proliferation in adult neurogenesis. Furthermore, this study analyzed only one point at 21 days; therefore, further research into time-dependent changes could clarify the pathophysiology of CO encephalopathy in greater detail. Moreover, in the present study, only the parallel changes of immunohistochemistry, flow-cytometry, and mRNA expression in adult neurogenesis were shown, but the correlations between the results could not be shown. These are the limitations of this study.

In conclusion, the results of the present study showed that CO exposure decreased the numbers of multipotent neural precursor cells, NG2^+^ oligodendrocyte precursor cells, and microglial cells in the adult DG, even after 21 days in our rat model. CO exposure also decreased the expression of neurogenic factors such as FGF2 and GDNF. The finding that involvement of adult hippocampal neurogenesis, especially multipotent neural precursor cells, could be greater in the pathophysiology of delayed CO encephalopathy is the novel point of the present study. In addition, the present results suggest a strategy for the development of a method to prevent and treat the cognitive impairment associated with delayed CO encephalopathy; antidepressants such as TCAs, SSRIs, and SNRIs may be useful by increasing FGF2 expression in astrocytes. To test this hypothesis, further investigation of the mechanisms underlying the effects of CO exposure on decreasing the number of neural precursor cells, NG2 cells, and microglial cells is needed.

## Materials and methods

### Experimental animals

All experiments were approved by the Animal Experiment Committee of Ehime University and conducted according to the Guidelines for Animal Experimentation of Ehime University Graduate School of Medicine (Ehime, Japan). This study was carried out in compliance with the ARRIVE guidelines (http://www.nc3rs.org.uk/page.asp?id=1357).

Male Wistar rats (5 weeks old) purchased from CLEA Japan (Tokyo, Japan) were housed three per plastic cage (ambient temperature 22 ± 2 °C), with a 12-h light–dark cycle and free access to food and water.

### Exposure of rats to CO

CO exposure was performed as described in our previous studies^10, 11, 16^. Briefly, 78 male Wistar rats (6 weeks old) were randomly divided into the control group (n = 39) and the CO-exposed group (n = 39). Rats were placed in a 7.6-L chamber (250 × 190 × 160 mm) (KN-1010-L, Natsume Seisakusho, Tokyo, Japan) and exposed to 1000 ppm CO for 40 min and then 3000 ppm for 20 min until they lost consciousness. In addition, rats that did not lose consciousness in this period were exposed to 10,000 ppm until they lost consciousness. Rats were then moved to an air-conditioned room to regain consciousness. Controls were exposed to room air for 60 min in the same chamber. For the duration of experiments, the concentrations of CO, CO_2_, and O_2_ were monitored.

**Figure 6:**
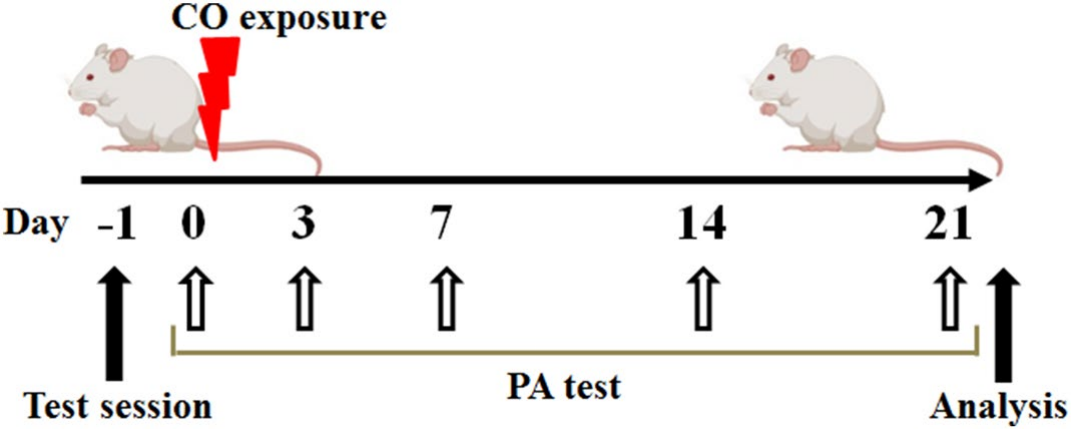
Time line of this study. The rats are received in the training session as the passive-avoidance (PA) test (Day -1) and are measured without foot-shocks 24 h later (Day 0). Then, rats are exposed to CO, and the latencies are measured at days 3, 7, 14, and 21 after exposure. The analyses of immunohistochemistry, flow cytometry, and quantitative reverse transcription-PCR are performed on day 21 after CO poisoning. This figure was created with BioRender.com.

The outline of this study is shown in Fig. 6.

### Passive-avoidance test

Behavioral effects on learning and memory function were measured with the step-through passive-avoidance test in control and CO-exposed rats until 21 days, as in our previous study^11^. Briefly, the apparatus consisted of an illuminated (450 × 270 × 260 mm^3^) compartment and a dark (450 × 270 × 260 mm^3^) compartment on a grid floor, separated by a sliding door (MPB-M001; Melquest, Toyama, Japan). Before CO exposure, each rat was placed in the illuminated compartment and allowed to explore for 20 s as the training session. The sliding door was then opened, and the step-through latency was measured for rats to enter the dark compartment with all four paws. The sliding door was immediately closed upon entry into the dark compartment. At least two additional trials were conducted, each separated by 5 min, until rats entered the dark compartment within 20 s. After the door was closed in the last trial, an electric foot-shock (1 mA for 5 s) was delivered through the grid floor with a constant current shock generator (SG-100; Melquest). The rats entered the dark compartment within 300 s in the training session, and the same foot-shocks were repeated until they did not enter the dark compartment within 300 s (Day -1). Rats were measured without foot-shocks 24 h after the training session (Day 0), and then rats were treated with room air or CO. The latencies were measured at 3, 7, 14, and 21 days after exposure. The step-through latency was recorded as “300 s” when rats did not enter the dark room after more than 300 s.

### Immunohistochemistry (IHC)

IHC was performed to determine the effects of CO exposure on neural precursor cells and glial cells in the adult DG, as described in our previous study^16^. Briefly, rats were sacrificed under anesthesia and perfused transcardially with 4% paraformaldehyde in phosphate-buffered saline (PBS) containing 2 mM MgCl_2_ 21 days after CO exposure. Whole brains were excised and immersed in PBS containing 20% sucrose overnight, and then the brains were rapidly mounted in optimal cutting temperature compound and frozen in dry ice. Brains were sliced serially into 10-µm-thick coronal sections using a cryostat. The sections were rinsed with Tris-buffered saline and then permeabilized and blocked with Tris-buffered saline containing 0.1% Tween 20 and 1 mg/ml bovine serum albumin. The sections were incubated with primary antibodies overnight at 4 °C. The primary antibodies used were mouse anti-SRY (sex-determining region Y)-box 2 (SOX2, 1:500; Abcam, Cambridge, UK), mouse anti-CD11b (1:500; Merck Millipore, Burlington, MA, USA), goat anti-GFAP (1:500; Abcam), rabbit anti-neuron glial antigen 2 (NG2, 1:500; Merck Millipore), and rabbit anti-brain lipid binding protein (BLBP, 1:500; Abcam). SOX2, BLBP, and GFAP were used to identify neural precursor cells and astrocytes. CD11b was used to identify microglial cells. NG2 was used to identify oligodendrocyte precursor cells. The immunoreaction was visualized using Alexa Fluor 488- (Thermo Fisher Scientific, Tokyo, Japan), Alexa Fluor 594- (Thermo Fisher Scientific), and Cy5- (Jackson Immuno Research Laboratories, West Grove, PA, USA) labeled secondary antibodies. For nuclear counter-staining, ProLong Gold Antifade Mountant with 4′,6-diamidino-2-phenylindole, dihydrochloride (DAPI) (Thermo Fisher Scientific) was used. We observed the sections using a BZ-9000 scanning fluorescence microscope (Keyence, Osaka, Japan), and SOX2^+^/BLBP^+^/GFAP^+^ cells, SOX2^+^/GFAP^−^ cells, SOX2^−^/GFAP^+^ cells, CD11b^+^ cells, and NG2^+^ cells were counted. SOX2^+^/BLBP^+^/GFAP^+^ cells and SOX2^+^/GFAP^−^cells were counted in the granular cell layer and subgranular zone in the DG, because neurogenesis occurs in the adult hippocampus in these subregions. On the other hand, SOX2^−^/GFAP^+^ cells, CD11b^+^ cells, and NG2^+^ cells were counted in the whole DG. Cell counting using a previously reported method^34^ was performed blinded with respect to the experimental group. Two sections of DG per rat (n = 8) were observed, and the total number of cells was obtained by counting DAPI-stained nuclei.

### Flow cytometry (FCM)

FCM was performed to determine the counts of microglial cells in the regions of the hippocampus, as described in our previous studies^16, 35^. Briefly, rats were sacrificed under anesthesia and perfused transcardially with PBS for 3 min to remove blood. Then, the bilateral hippocampal tissues were excised. According to the manufacturer’s protocol, hippocampal tissues were dissociated into single cells using a gentle MACS dissociator (Miltenyi Biotec, Tokyo, Japan) and the adult brain dissociation kit (Miltenyi Biotec), and undissociated tissues were removed using MACS Smart Strainers with 100-μm pores (Miltenyi Biotec). Then, debris and erythrocytes were removed using the debris removal and red blood cell removal solutions contained in the adult brain dissociation kit (Miltenyi Biotec). The prepared cell suspensions were subjected to FCM analyses. The single-cell suspensions were diluted to 1 × 10^6^ cells/100 μl with PBS containing 2 mM ethylenediaminetetraacetic acid and 2% fetal bovine serum. To block Fc receptors, the single cells were incubated with an anti-CD32 antibody Fc blocker (BD Pharmingen, Franklin Lakes, NJ, USA) for 20 min on ice.

The single cells were incubated with fluorescence-labeled antibodies for 30 min on ice. The fluorescence-labeled antibodies were CD11b/c-PE/Cy7 (1:100; BD Pharmingen), CD45-PE (1:100; BioLegend, San Diego, CA, USA), and Granulocytes-FITC (1:100; Miltenyi Biotec). The single cells labeled with antibodies were analyzed on a Gallios flow cytometer (Beckman Coulter, Tokyo, Japan). Zombie NIR (BioLegend) was used for the analyses of cell viability. Data analyses were performed with Flow Jo Software version 7.6.5 (Tree Star, Inc., Ashland, OR, USA).

### Total RNA isolation and quantitative reverse transcription-PCR (RT-PCR) analysis of gene expression

Rats were sacrificed under anesthesia at 21 days after CO exposure, and bilateral hippocampal tissues were excised on an ice-cold stage. Total RNA samples were obtained from hippocampal tissues using the RNeasy kit (Qiagen, Valencia, CA, USA), according to the manufacturer’s protocol. The RNA quantity was measured with NanoDrop-1000 (Thermo Fisher Scientific), and the RNA integrity was measured with an Agilent 2100 Bioanalyzer (Agilent Technologies, Loveland, CO, USA). RNA was reverse transcribed to cDNA using the High Capacity cDNA Reverse Transcription Kit (Applied Biosystems, Foster City, CA, USA). For analysis of mRNA expression levels, comparative evaluation of quantitative RT-PCR was performed using the TaqMan gene expression master mix and the StepOnePlus Real-Time PCR System (Applied Biosystems). The assay IDs of TaqMan probes (Applied Biosystems) were Rn00570809_m1 for fibroblast growth factor 2 (*Fgf2*), Rn01402432_m1 for glial cell-derived neurotrophic factor (*Gdnf*), Rn01484924_m1 for brain-derived neurotrophic factor (*Bdnf*), and Rn99999916_s1 for glyceraldehyde-3-phosphate dehydrogenase (*Gapdh*). *Gapdh* was used as an internal standard. mRNA expression in CO rats and controls was examined with the average of triplicate measurements. The ΔΔCt method was used to quantify the relative expression levels.

### Statistical analysis

Statistical analyses were performed with SPSS 23.0 software (IBM Japan, Tokyo, Japan). The Shapiro–Wilk test was used as a test of normality. Student’s *t*-test or the Mann–Whitney U test was used for comparisons between control and CO groups. Descriptive statistics are expressed as mean ± standard error of the mean (SEM) in the figures, and a *p* value less than 0.05 was considered significant.

## Supplementary Information

Supplementary Figure 1.

Supplementary Legend.
