# Supplementary figures and images for: Neural precursor cells are decreased in the hippocampus of the delayed carbon monoxide encephalopathy rat model
Source: Sci Rep. 2021 Mar 18;11:6244. doi: 10.1038/s41598-021-85860-9 (PMC7973557; doi:10.1038/s41598-021-85860-9)

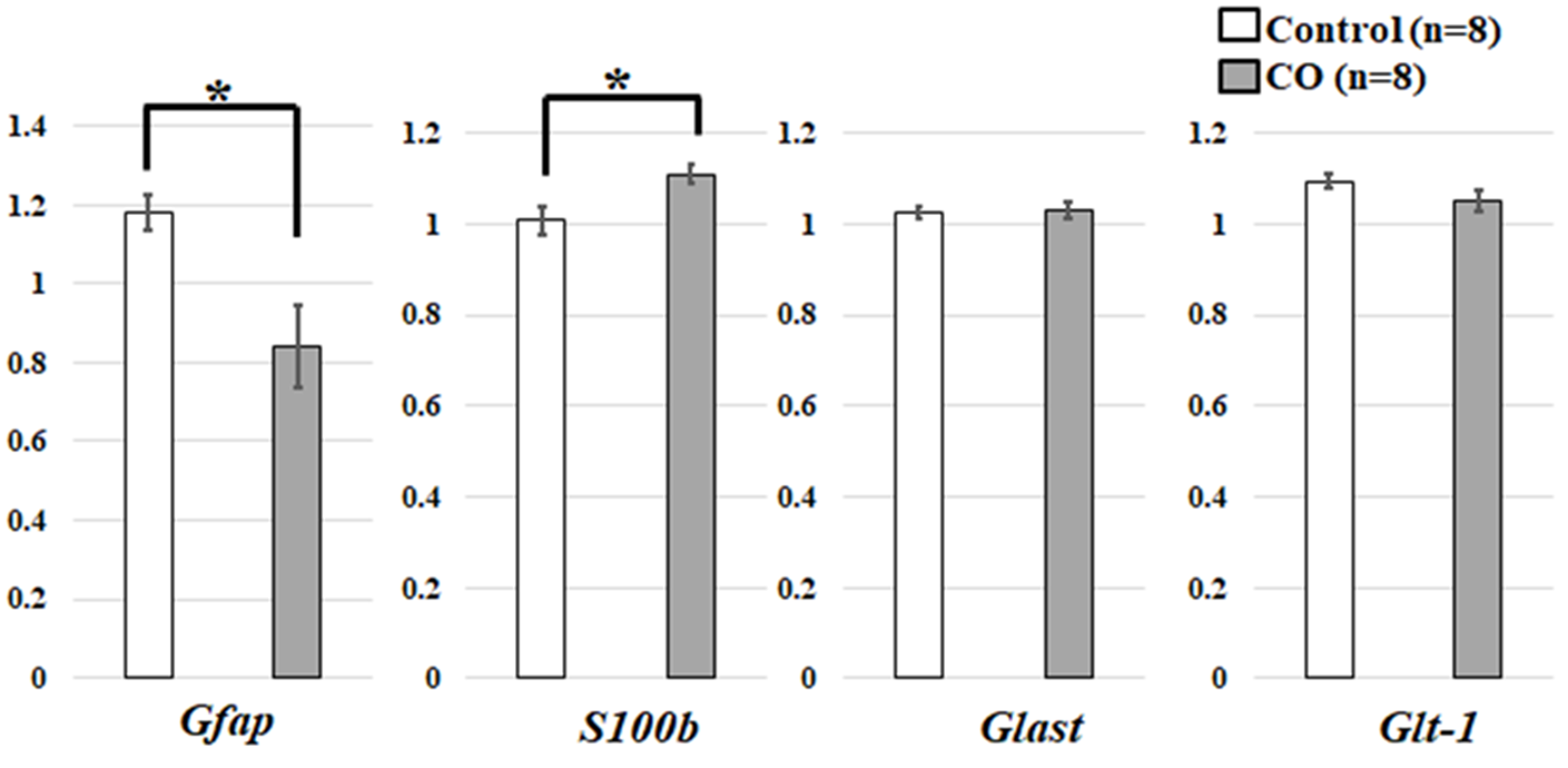

Supplement: Supplementary file 1 — Supplementary Figure 1. [file 41598_2021_85860_MOESM1_ESM.tif]
