# Supplementary material for: Neural precursor cells are decreased in the hippocampus of the delayed carbon monoxide encephalopathy rat model
Source: Sci Rep. 2021 Mar 18;11:6244. doi: 10.1038/s41598-021-85860-9 (PMC7973557; doi:10.1038/s41598-021-85860-9)
Supplement: Supplementary file 2 — Supplementary Legend. [file 41598_2021_85860_MOESM2_ESM.docx]

Supplemental Figure 1. mRNA expression in astrocytes.

The mRNA expression of *Gfap* is significantly lower and that of *S100β* is significantly higher in the CO rats 21 days after CO exposure than in the controls. The mRNA expression of *Glt-1* and *Glast* is not significantly different in the CO rats 21 days after CO exposure. Values are expressed as mean ± SEM. **p* < 0.05.
